# DIFFERENCES IN PROTECTIVE MENTAL HEALTH RESOURCES AMONG WIDOWED IN THE UNITED STATES AND INDIA

**DOI:** 10.1093/geroni/igae098.1664

**Published:** 2024-12-31

**Authors:** Shekhar Chauhan, Dawn Carr, Miles Taylor, Amanda Sonnega

**Affiliations:** Florida State University, Tallahassee, Florida, United States; Florida State University, Tallahassee, Florida, United States; Florida State University, Tallahassee, Florida, United States; University of Michigan, University of Michigan, Michigan, United States

## Abstract

Widowhood is among the most consequential stressful events for mental health. Although social support is protective for mental health at widowhood, most of widowhood studies are based on US populations. This study uses a harmonized dataset we created to evaluate differences in depressive symptoms among recently widowed individuals (i.e., within the last 2 years) in the United States and India using the first (2017-18) wave of the Longitudinal Ageing Study in India (LASI; n=1571) and the 2016-2018 waves of the Health and Retirement Study (n=202). Using OLS regression, we evaluate depressive symptoms based on a four-item depression instrument (CESD; mean=1.19), by country status and we use interaction effects to evaluate differences by country. Results show that mean depressive symptoms are lower among US widows (0.95 vs 1.22). Having children and higher relative socioeconomic status are both associated with protective effects for US and Indian widowed people, but having friends and rural status are only associated with lower depressive symptoms among US widows. Rural status is associated with higher symptoms in India. Gender is not significant in either sample. Self-rated health is associated with protective effects for depressive symptoms for both groups, but the effect is greater for US widows. Findings suggest there are certain protective resources that are consistent across contexts, especially self-rated health, socioeconomic status and having children. Mental health interventions for widows that focus on non-family social engagement, however, may only benefit recent widows in the US.

